# Triiodothyronine levels in athyreotic pediatric patients during levothyroxine therapy

**DOI:** 10.3389/fendo.2024.1443394

**Published:** 2024-08-14

**Authors:** Julia Baran, Amber Isaza, Mya Bojarsky, Lama Alzoebie, Minkeun Song, Stephen Halada, Lindsay Sisko, Stephanie Gonzales, Sogol Mostoufi-Moab, Andrew J. Bauer

**Affiliations:** ^1^ Division of Endocrinology and Diabetes, Children’s Hospital of Philadelphia, Philadelphia, PA, United States; ^2^ Division of Oncology, Children’s Hospital of Philadelphia, Philadelphia, PA, United States

**Keywords:** combination therapy, T_4_ monotherapy, levothyroxine, hypothyroidism, pediatrics, liothyronine

## Abstract

**Objective:**

Levothyroxine (LT_4_) monotherapy is the current recommended approach for treating pediatric patients post-total thyroidectomy (TT) based on the assumption that peripheral conversion of thyroxine (T_4_) to triiodothyronine (T_3_) normalizes thyroid hormone levels. In adults, approximately 15% of post-TT patients on LT4 monotherapy have altered T_4_:T_3_ ratios with ongoing debate in regard to the clinical impact with respect to health-related quality of life (hrQOL). The ability to normalize T_3_ and T_4_ levels on LT_4_ monotherapy for pediatric patients’ post-TT is important but not previously described. This study reports data on T_3_ levels in athyreotic pediatric patients to determine if a similar cohort of patients exists on LT4 monotherapy targeting normalization of TSH (LT4 replacement) or suppression (LT4 suppression).

**Methods:**

Thyroid function tests (TFTs) were retrospectively extracted from medical charts for patients <19 years old who underwent TT for definitive treatment of Graves’ disease (GD) or differentiated thyroid cancer (DTC) between 2010–2021. LT4 dosing was selected to normalize the TSH in GD patients (LT4 replacement) or suppress TSH in DTC patients (LT4 suppression). Pre- and post-surgical TSH, T3 and T4 levels were compared.

**Results:**

Of 108 patients on LT_4_ replacement (n=53) or LT_4_ suppression (n=55) therapy, 94% (102/108) of patients demonstrated T_3_ levels in the normal range post-TT. However, the majority of patients on LT_4_ replacement (44/53; 83%) and LT_4_ suppression (31/55; 56%) displayed post-TT T_3_ levels in the lower half of the normal range despite 50% (22/44) and 48% (15/31) of these patients, respectively, having post-TT fT_4_ levels above the upper limit of the normal range.

**Conclusion:**

A significant number of pediatric patients do not achieve similar T_3_ and T_4_:T_3_ levels pre- and post-TT. Future multi-center, prospective studies evaluating LT_4_ monotherapy in comparison to combined LT_4_/LT_3_ therapy are warranted to determine the potential clinical impact of altered T3 levels in athyreotic pediatric patients.

## Introduction

The thyroid gland is responsible for secreting thyroxine (T_4_) and triiodothyronine (T_3_), hormones that influence growth, neurocognitive development and function, metabolism, and mood (1–3). Under tissue-specific regulation and expression of type 1 and type 2 deiodinases, peripheral conversion of the pro-hormone thyroxine (T_4_) to the active hormone triiodothyronine (T_3_) accounts for 70–80% of serum T_3_ concentrations (4, 5). For several decades, levothyroxine (LT_4_) monotherapy has been the standard approach to care for patients who have undergone total thyroidectomy (TT). This treatment is based on the assumption that peripheral conversion of T_4_ to T_3_ is sufficient to achieve normal serum and tissue levels and that thyroid stimulating hormone (TSH) is the most sensitive and specific marker of hypothalamic-pituitary-thyroid axis homeostasis (6, 7). While LT_4_ monotherapy can normalize serum TSH in athyreotic patients, it may generate low circulating levels of T_3_ and increased T_4_:T_3_ ratios (8). In adults, data demonstrate that even when LT_4_ is prescribed to target TSH and T_4_ levels within or above the normal range, up to 15% of patients have T_3_ levels at or below the lower limit of normal (LLN) (9). A current area of investigation in adults is focused on whether T3 levels on LT_4_ monotherapy are associated with (1) persistent symptoms, including fatigue, weight gain, depressed mood, (2) decreased health-related quality of life (hrQOL), and/or (3) treatment dissatisfaction (10, 11).

Liothyronine (LT_3_) combined with LT_4_ therapy is an alternative treatment option for hypothyroidism in athyreotic adult patients as it can be tailored to improve T_3_ levels and T_4_:T_3_ ratios compared to LT_4_ alone (12). There are mixed data in adults regarding whether combined therapy is effective at improving hrQOL, with several studies demonstrating a positive impact (13, 14) and others showing no advantage in using combination therapy to improve hrQOL compared to LT_4_ monotherapy (8, 15–18). While an ongoing discussion persists regarding best practices to treat post-TT hypothyroidism in adults, the applicability of LT_4_ monotherapy to normalize thyroid hormone levels in athyreotic children and adolescents is equally important but not previously described. This is the first study to evaluate T_3_ levels in athyreotic pediatric patients on LT_4_ monotherapy to determine whether patients achieve T_3_ normalization and if post-TT thyroid hormone levels are comparable to baseline, pre-operative thyroid hormone levels.

## Methods

### Selection criteria and cohort

A retrospective chart review was conducted of patients who underwent TT for GD or DTC between January 2010-December 2021 at the Children’s Hospital of Philadelphia. Patients were selected if they had complete TFTs, including a TSH, T3 and free T4 (free T4) prior to surgery and 12 ± 6 months post-TT. The mean treatment duration before TFT testing was 12 ± 3 months. A final cohort of 108 patients meeting the eligibility criteria were included in the analysis, 53 GD patients on LT_4_ replacement and 55 DTC patients on LT_4_ suppression post-TT. Patient demographics, medication history, surgical approach, clinical symptoms, and thyroid function tests (TFTs) were collected. TFTs were drawn at CLIA-certified labs and evaluated in accordance with insurance capitation and proximity to the patient’s home or primary care institution. LT_4_ was dosed to target normalization of TSH (LT4 replacement with TSH in the normal range, between 0.5–4.5 μIU/L) or suppression of TSH (LT4 suppression with TSH <0.5 μIU/L) after TT for GD or DTC, respectively, based on current clinical guidelines (19, 20). TFTs were obtained at various times throughout the day in a non-fasting state.

### Statistical analysis

Continuous variables were summarized by mean ± standard deviation for parametric data and median (IQR) for nonparametric data. Categorical variables were summarized by frequency and percent. Due to the retrospective nature of this study with associated variance in laboratory assays used, fT_4_ and T_3_ values were grouped into four reference interval categories: below the limit of the normal range, in the lower half of the normal range, in the upper half of the normal range, and above the limit of the normal range. Pre- and post-TT fT_4_:T_3_ ratios [(fT_4_*100) ÷ T_3_] and TFTs were compared for both cohorts using paired t-test. The fT_4_:T_3_ ratio was adopted from Jonklaas et al. (21). *P*-values ≤0.05 were considered statistically significant. All analyses were performed in JMP Pro 16.

## Results

### Demographics

Demographics of 53 GD patients on LT_4_ replacement and 55 DTC patients on LT_4_ suppression are summarized in **Table 1**. Patients underwent TT at a median age 14.7 years (IQR=13.0–16.6). Female sex was predominant in both cohorts consistent with the prevalence of autoimmune thyroid disease and DTC in adolescent girls. There was no statistical difference in the mean with SD or median with IQR in the timing for pre-operative and post-operative TFTs, both approximately one month prior to surgery and one year after surgery.

**Table 1:** Demographic and clinical characteristics of pediatric patients on LT_4_ monotherapy following thyroidectomy.

	Total N = 108	GD N = 53	DTC N = 55
Demographics, N (%)			
Sex			
Male	23 (21.3)	9 (17.0)	14 (25.5)
Female	85 (78.7)	44 (83.0)	41 (74.5)
Race			
Asian	8 (7.4)	4 (7.5)	4 (7.3)
Black or African American	8 (7.4)	7 (13.2)	1 (1.8)
White	76 (70.4)	36 (67.9)	40 (72.7)
Other or Not Reported	16 (14.8)	6 (11.3)	10 (18.1)
Ethnicity			
Hispanic or Latino	12 (11.1)	4 (7.5)	8 (14.5)
Not Hispanic or Latino	93 (86.1)	47 (88.7)	46 (83.6)
Unknown or Not Reported	3 (2.8)	2 (3.8)	1 (1.8)
Age at Surgery			
Mean (SD)	14.4 (2.9)	14.4 (3.0)	14.4 (2.9)
Median (IQR)	14.7 (13.0–16.6)	14.8 (13.5–16.4)	14.6 (12.3–16.7)
TFT Collection, days			
Preoperative TFTs			
Mean (SD)	28 (41)	30 (52)	27 (28)
Median (IQR)	18 (9–31)	16 (9–26)	19 (10–33)
Postoperative TFTs, days			
Mean (SD)	365 (87)	361 (93)	369 (82)
Median (IQR)	364 (303–406)	349 (298–431)	369 (321–403)

GD, Graves’ Disease; DTC, Differentiated Thyroid Cancer; TFT, Thyroid Function Test.

### TFT concentrations pre- and post-total thyroidectomy

**Figure 1:**
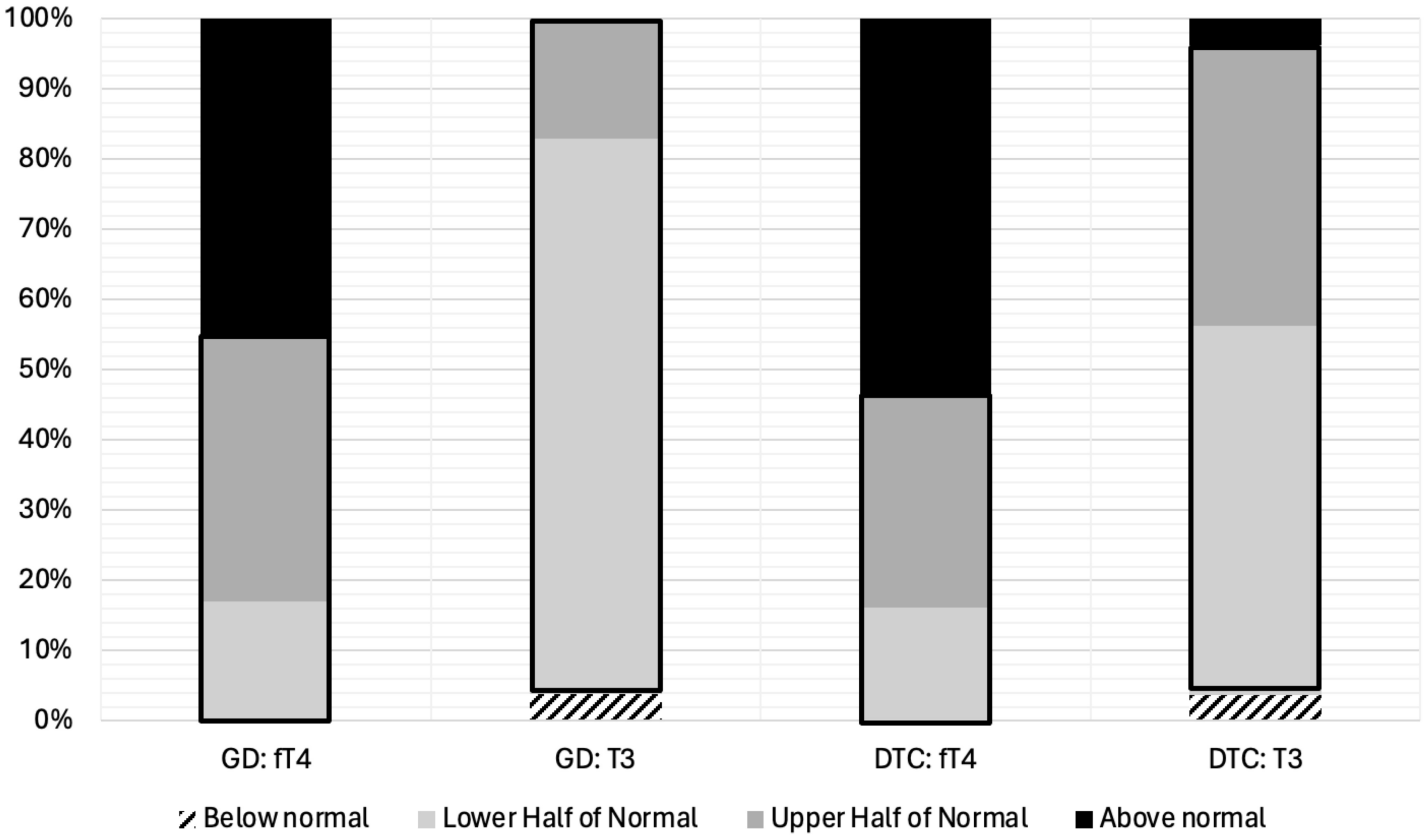
fT_4_ and T_3_ Levels Stratified by Quartiles for Pediatric Patients on LT_4_ Monotherapy Post-Thyroidectomy for Treatment of Graves’ Disease (N=53) or Differentiated Thyroid Cancer (N=55). Thyroid hormone levels have been divided into four categories; (1) below normal, (2) lower half of the normal range, (3) upper half of the normal range, and (4) above the normal range. The normal range is outlined in black. GD, Graves’ Disease; DTC, Differentiated Thyroid Cancer; fT_4_, free Thyroxine; T_3_, Triiodothyronine.

From the total cohort, ninety four percent of patients (102/108; 94%) demonstrated post-TT T_3_ levels within the normal range. However, the majority of patients on LT_4_ replacement (44/53; 83%) or LT_4_ suppression (31/55; 56%) displayed post-TT T_3_ levels in the lower half or below the normal range despite LT4 dosing targeted to have post-TT fT4 in the upper half (GD) or above the upper limit (DTC) of the normal range (**Figure 1**).

**Figure 2:**
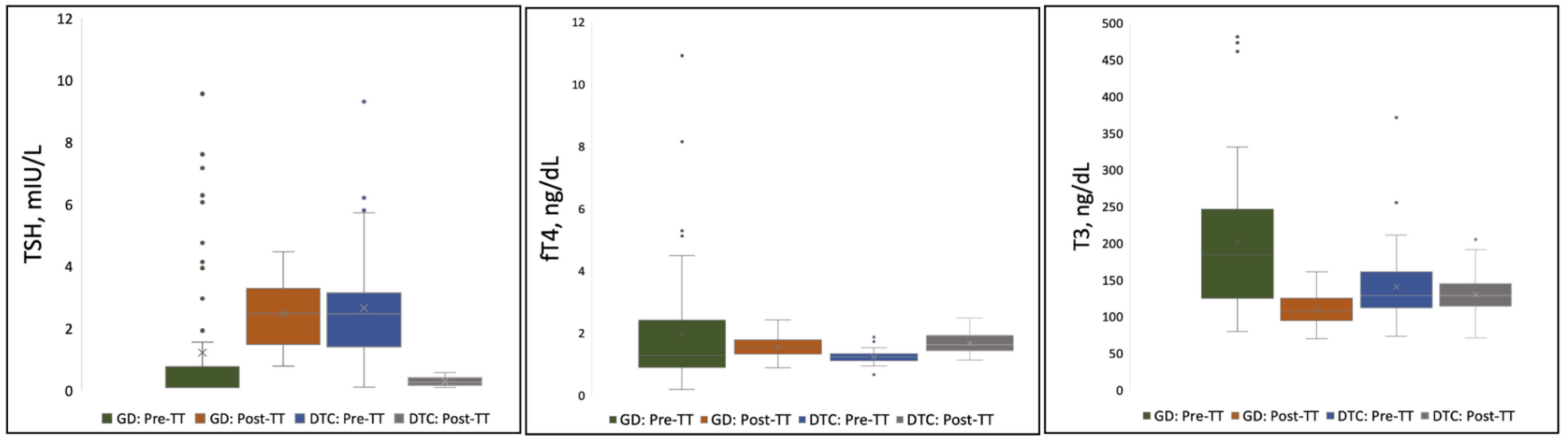
Distribution of TSH, fT_4_, and T_3_ Concentrations Pre- and Post-Thyroidectomy for Pediatric Patients on LT_4_ Monotherapy. Each shaded box represents data within the 25^th^-75^th^ percentile. Lines extending from the shaded box represent data within the 5^th^-95^th^ percentile. Values (point markings) above and below the 5^th^ and 95% line represent outliers. “X”in the center of each box represents the arithmetic mean. GD, Graves’ Disease; DTC, Differentiated Thyroid Cancer; TSH, Thyroid Stimulating Hormone; fT_4_, free Thyroxine; T_3_, Triiodothyronine; TT, Total Thyroidectomy.

TSH, T3, and fT_4_ concentrations measured pre- and post-TT for GD and DTC patients are presented in **Table 2** and **Figure 2**. In GD patients, the pre-TT T3 levels were above the normal range with a significant decrease in mean T_3_ concentration observed post-TT with LT4 dosed to normalize the TSH (LT4 replacement therapy, 108 ng/dL, 95% CI=103–114, 235 ng/dL, 95% CI=181–290(p<0.0001), respectively. In contrast, in DTC patients with normal pre-TT T3 and LT4 dosed TSH suppression, there was no significant decrease in mean T_3_ concentration pre- and post-TT, 139 ng/dL, 95% CI=127–151 compared to 129 ng/dL, 95% CI=122–136 (p=0.083), respectively. Mean TSH and fT4 concentrations were normal with no significant difference for patients with GD (**Table 2**). In DTC patients, with LT4 suppressive therapy, TSH was significantly lower with associated significantly higher fT4 (**Table 2**).

**Table 2:** TSH, fT_4_, and T_3_ concentrations pre- and post-thyroidectomy for pediatric patients on LT_4_ monotherapy.

	GD N = 53			DTC N = 55		
	Pre-TT	Post-TT	P-Value	Pre-TT	Post-TT	P-Value
TFT*						
TSH, mIU/L						
Mean (SD)	1.51 (3.57)	2.41 (1.08)	0.101	2.60 (1.68)	0.21 (0.15)	<0.0001
[95% CI]	0.55–2.47	2.12–2.70		2.15–3.04	0.17–0.24	
fT_4_, ng/dL						
Mean (SD)	1.97 (1.91)	1.52 (0.31)	0.098	1.19 (0.20)	1.64 (0.31)	<0.0001
[95% CI]	1.45–2.48	1.44–1.60		1.14–1.24	1.56–1.72	
T_3_, ng/dL						
Mean (SD)	235.0 (170.6)	108.4 (19.9)	<0.0001	139.3 (45.9)	129.1 (27.8)	0.08
[95% CI]	180.5–289.5	103.1–113.8		127.2–151.4	121.7–136.4	
Ratio [(fT4*100)/T3						
Mean (SD)	0.82 (0.41)	1.44 (0.34)	<0.0001	0.92 (0.26)	1.33 (0.36)	<0.0001
[95% CI]	0.71–0.93	1.35–1.53		0.85–0.99	1.23–1.42	

*Reference ranges for TSH, fT_4_, and T_3_ were collected from the assay performed. The mean treatment duration post-TT was 1 year ± 3 months.

GD, Graves’ Disease; DTC, Differentiated Thyroid Cancer; TFT, Thyroid Function Test; TSH, Thyroid Stimulating Hormone; fT_4_, free Thyroxine; T_3_, Triiodothyronine; TT, Total Thyroidectomy.

Mean fT_4_:T_3_ ratios for both the GD and DTC cohorts, however, were significantly higher post-TT compared to pre-TT (p<0.0001; **Table 2**), reflecting the majority of patients having a T3 in the lower-half or below the normal range despite high normal to elevated fT4 on LT4 replacement (GD) or suppressive (DTC) therapy. Mean pre- and post-TT fT_4_:T_3_ ratios for GD patients were 0.82 (SD=0.41; range=0.17–2.61) and 1.44 (SD=0.34; range=0.68–2.30), respectively. Mean pre- and post-TT fT_4_:T_3_ ratios for DTC patients were 0.92 (SD=0.26; range=0.37–1.53) and 1.33 (SD=0.36; range=0.66–2.59), respectively. Expectedly, post-TT T_3_ and fT_4_ levels were higher in patients on LT_4_ suppression compared to patients on LT_4_ replacement (T_3_ p<0.0001; fT_4_ p=0.046).

## Discussion

We evaluated the utility of LT_4_ monotherapy to normalize thyroid hormone levels in a cohort of pediatric patients that underwent thyroidectomy for the treatment of GD or DTC. majority of patients (102/108; 94%) achieved T_3_ normalization post-TT, 69% (75/108) of patients demonstrated T_3_ levels in the lower half or below the normal range despite having fT4 in the upper half or above the normal range (**Figure 1**). The high percentage of patients with an increase in mean fT_4_:T_3_ ratio post-TT compared to fT_4_:T_3_ ratio pre-TT for both cohorts suggests that peripheral deiodination of exogenous LT_4_ may be insufficient in achieving similar T_4_ and T_3_ levels for some athyreotic pediatric patients. Our findings corroborate previous studies evaluating the efficacy of LT_4_ monotherapy to achieve normal T3 levels in the treatment of post-TT hypothyroidism in the adult population (8). In fact, our fT_4_:T_3_ ratios were comparable to those reported by Jonklaas et al. in 50 athyreotic adults (pre-TT/post-TT): 0.82/1.09 for GD patients and 0.91/1.27 for DTC patients (**Table 2**) (21).

While the implications of higher fT_4_:T_3_ ratios in post-TT patients treated with LT4 monotherapy is not well defined, future studies comparing LT4 monotherapy against LT_4_/LT_3_ combination therapy may be worthwhile in athyreotic pediatric patients with (1) low serum T_3_ concentrations and (2) who demonstrate persistent symptoms despite appropriate TSH in target on LT_4_ replacement or suppressive dosing. If one targets normalization of T3, T4 and TSH, there is no anticipated risk to combined LT_4_/LT_3_ therapy. The potential negative impact of combined therapy includes the additional cost to prescribing LT_3_ along and T3 surveillance labs and the need for multiple daily doses of LT_3_ secondary to the short serum half-life of current LT_3_ formulations. However, if patients achieve improved hrQOL, the benefit of combined therapy would be worth the additional cost and multi-daily dose schedule (22, 23). The use of combined LT_4_ and LT_3_ therapy in selected pediatric patients would be in keeping with the joint consensus statement from the American, British, and European Thyroid Associations (24) as well as other adult thyroidologists.

This study is limited by its single-center retrospective design and non-centralized laboratory assay quantification. In addition, there are no data on the clinical benefit of normalizing T3 in pediatric patients in regard to hrQOL, cardiovascular health, or, potentially, growth and development. In fact, the non-specific signs and symptoms of hypothyroidism and multiple confounding variables that impact fatigue, mood, caloric metabolism, and cardiovascular health have precluded completion of a multi-center, prospective study in the adult population secondary to the required cohort size and study cost. One would anticipate the same challenges for a potential, prospective study between LT4 monotherapy and LT4/LT3 combination therapy in pediatrics. Despite these limitations, this study is the first to evaluate T_3_ levels in athyreotic children and adolescents and provides valuable information that may further inform the pediatric thyroid community in on-going efforts to optimize thyroid hormone therapy management post-TT.

In an effort to optimize the evaluation of thyroid hormone replacement, future studies should also include analysis for single nucleotide polymorphisms of the deiodinase 2 gene, including Thr92Ala, that have previously been found to be associated with decreased T_4_ to T_3_ conversion in adults (25, 26), validated, patient-reported hrQOL instruments to assess patient satisfaction, and metabolomic analysis as a potential tool to more completely understand the impact of therapy on intracellular T3 levels (27, 28).

International consortia dedicated to collaborative efforts to improve patient care are critical to conducting these future studies. Accordingly, the authors have established the Child and Adolescent Thyroid Consortium, an international consortium that provides an infrastructure to conduct multi-center studies dedicated to pediatric thyroid disorders (www.thyroidcatc.org).

## Conclusion

Similar to adults, a significant number of pediatric patients do not achieve similar T_3_ and fT_4_:T_3_ levels on LT_4_ monotherapy pre- and post-TT. Future multi-center, prospective studies evaluating LT_4_ monotherapy in comparison to LT_4_/LT_3_ combination therapy are warranted to determine the potential clinical impact of altered T3 levels in athyreotic pediatric patients.

## Data Availability

The original contributions presented in the study are included in the article/supplementary material. Further inquiries can be directed to the corresponding author.
